# The metabolic overdrive hypothesis: hyperglycolysis and glutaminolysis in bipolar mania

**DOI:** 10.1038/s41380-024-02431-w

**Published:** 2024-01-25

**Authors:** Iain H. Campbell, Harry Campbell

**Affiliations:** 1 Division of Psychiatry, Centre for Clinical Brain Sciences, University of Edinburgh, Kennedy Tower, Royal Edinburgh Hospital, Edinburgh, EH10 5HF UK; 2 Usher Institute, Centre for Global Health Research, University of Edinburgh, Craigour House, 450 Old Dalkeith Rd, Edinburgh, EH16 4SS UK

**Keywords:** Bipolar disorder, Neuroscience

## Abstract

Evidence from diverse areas of research including chronobiology, metabolomics and magnetic resonance spectroscopy indicate that energy dysregulation is a central feature of bipolar disorder pathophysiology. In this paper, we propose that mania represents a condition of heightened cerebral energy metabolism facilitated by hyperglycolysis and glutaminolysis. When oxidative glucose metabolism becomes impaired in the brain, neurons can utilize glutamate as an alternative substrate to generate energy through oxidative phosphorylation. Glycolysis in astrocytes fuels the formation of denovo glutamate, which can be used as a mitochondrial fuel source in neurons via transamination to alpha-ketoglutarate and subsequent reductive carboxylation to replenish tricarboxylic acid cycle intermediates. Upregulation of glycolysis and glutaminolysis in this manner causes the brain to enter a state of heightened metabolism and excitatory activity which we propose to underlie the subjective experience of mania. Under normal conditions, this mechanism serves an adaptive function to transiently upregulate brain metabolism in response to acute energy demand. However, when recruited in the long term to counteract impaired oxidative metabolism it may become a pathological process. In this article, we develop these ideas in detail, present supporting evidence and propose this as a novel avenue of investigation to understand the biological basis for mania.

## Introduction: energy dysregulation in bipolar disorder

For over a century, cyclical energy dysregulation has been recognized as a core deficit in bipolar disorder (BD) and has been described as early as 1854 by Jean Pierre Falret in his seminal paper “la folie circulaire” [1]. Emil Kraepelin’s “Manic Depressive Illness and Paranoia”—the basis for modern classification of mental disorders—noted in 1921 that during bipolar depression “the total absence of energy is very specially conspicuous”. He also noted that during mania patients exhibit a “colossal energy for work” they are “a stranger to fatigue… activity goes on day and night” [2].

Since then, over a century of scientific research has substantiated these early clinical observations. Significant shifts in circadian rhythm, rest, movement, and psycho-motor activity indicate a dysregulation of energy generation and expenditure in bipolar patients [3, 4]. Metabolomics and imaging data indicate a significant role of mitochondrial dysfunction in bipolar disorder representing disruption of energy production at the cellular level [5].

Lived experience reports of bipolar disorder patients consistently report shifts in energy level as a central feature of the condition. When asked about the first noticeable indication of onset of mania, patients reported “change in energy level” more than any other symptom [6]. A recent network analysis of 486 patients noted low energy and high energy as the most significant symptoms predicting depression and mania, respectively. The study also noted that the majority of the other reported symptoms were predicted directly or indirectly by the presence of low and high energy [7].

People with bipolar disorder in manic, hypomanic, and mixed states engage in higher levels of physical exercise than those who are depressed. Many even feel a compulsion to exercise and will substantially increase their level of physical activity during mania [8–10]. Conversely, exhaustion and lack of energy are characteristic of bipolar depression. Akinetic, catatonic states of depression where movement and activity are severely limited are reported in as many as 28% of bipolar patients [11].

## Mechanisms contributing to cerebral energy dysregulation

Glucose metabolism and its regulatory mechanisms play a central role in energy (ATP) production in the body and brain by providing fuel for oxidative phosphorylation in mitochondria. Metabolic dysfunction characterized by abnormal glucose metabolism, mitochondrial dysfunction, and insulin resistance (IR) are increasingly recognized as important features in the pathophysiology of bipolar disorder [12, 13].

Generation of ATP in mitochondria depends largely on oxidative phosphorylation driven by glucose metabolism. Evidence from magnetic resonance spectroscopy [MRS], positron emission tomography [PET] and blood metabolomics indicate that glucose metabolism is impaired or dysregulated in bipolar disorder [14].

Cerebral glucose metabolism involves several interrelated processes by which the brain derives energy from glucose. These include glucose uptake through the blood-brain barrier, glycolysis, which breaks down glucose into pyruvate while producing energy in the form of ATP; and oxidative phosphorylation, wherein pyruvate is converted to acetyl-CoA, which enters the citric acid cycle, driving the generation of ATP in the electron transport chain. The primary features of dysregulated glucose metabolism observed in bipolar disorder are impairment of oxidative phosphorylation -which accounts for the most significant part of glucose metabolism under normal conditions- and upregulation of glycolysis [15].

Glycolysis is a less efficient process than oxidative phosphorylation, producing only 2 ATP per glucose molecule compared with up to 36 ATP from oxidative phosphorylation [16]. However, glycolysis can metabolize glucose at a much faster rate allowing the brain the enter states of hypermetabolism. Long-term upregulation of glycolysis in response to impaired oxidative phosphorylation represents a pathological adaptation occurring in conditions of mitochondrial dysfunction.

The cause of dysregulated glucose metabolism in bipolar is not known, however endocrinological dysfunction such as hyperinsulinemia may apply an allostatic load which initiates and/or accelerates the course of illness in those who are genetically susceptible. For example, people with type 2 diabetes or insulin resistance have three fold higher odds of a chronic course of illness compared to those with normal glycemia [12].

Impaired cerebral glucose metabolism has also been observed in unipolar depression [17], and may therefore represent a marker of energy deficit common to several types of depression. However, mood- related dysregulation of cerebral metabolism, rather than simply chronic impairment, appears to characterize bipolar disorder. Research comparing cerebral metabolic rate across mood states (depressed, euthymic and manic) in bipolar disorder and unipolar depression (depressed & euthymic states) reported a significant increase in metabolic rate when moving from bipolar depression into the euthymic or manic state [18]. And no significant overall change in unipolar depression when moving from the depressed to euthymic state. Although there is little research measuring cerebral glucose metabolism intensively across bipolar mood states, in this study, metabolic rate was also measured across time in a rapid cycling bipolar patient. Average whole brain metabolic rates were 36% higher on hypomanic days than depressed suggesting a dynamic dysregulation of cerebral metabolism linked to mood state, rather than a state of static impairment [18]. The more variable, and perhaps mood-dependent, nature of cerebral metabolism in bipolar disorder may implicate dysfunction in dynamic mechanisms regulating glucose metabolism.

We have proposed that these metabolic features of bipolar disorder have a common root in dysfunction of insulin-signaling mechanisms, resulting in alternating states of glucose hypometabolism and hypermetabolism in the brain [19]. We have recently highlighted evidence, drawing on over 70 years of research, that Lithium, directly and indirectly, targets several key nodes of the phosphatidylinositol 3–kinase (PI3K)/protein kinase B (Akt) insulin signaling pathway, including GSKβ, the phosphatidylinositol cycle (PI-Cycle), Akt, protein kinase C and sodium-myo-inositol transporter (SMIT). GSKβ, a serine/threonine protein kinase, has been a central focus of bipolar research for the past 60 years [20]. GSK3β activity influences both glucose metabolism and circadian clock entrainment in the suprachiasmatic nucleus [21, 22].

Central to the PI3K pathway is the PI-cycle, which is another of the most researched mechanisms in bipolar disorder [23]. For several decades PI-cycle inhibition has been a target of substantial interest to pharmaceutical research and has been considered a competing hypothesis to GSK3β inhibition for the primary mechanism of action of Lithium. We have highlighted evidence that both the PI-cycle and GSK3β play critical roles in the underlying insulin signaling mechanism.

In this paper, we propose that there may be a metabolic phenotype of bipolar disorder where dysregulation of normal oxidative glucose metabolism causes the brain to oscillate between states of glucose hypo-metabolism (experienced subjectively as depression) and states of hyperglycolysis and upregulation of glutaminolysis (experienced subjectively as [hypo]mania). In this paper, we present evidence from other conditions in which mania is found as an ancillary finding, that the states of hyperglycolysis and glutaminolysis may serve as a biological basis of mania.

## Evidence for hyperglycolysis and altered glutamate metabolism in bipolar disorder

MRS studies have established increased brain glutamate as a central feature of bipolar disorder pathophysiology. Research investigating the role of glutamate in bipolar disorder has primarily focused on its role as an excitatory neurotransmitter. However, we have proposed that it may have a further important role in bipolar disorder, acting as an energy substrate for the tricarboxylic acid (TCA) cycle through glutaminolysis, where it is converted to TCA cycle intermediate alpha-ketoglutarate. Indeed, the role of glutamate as a metabolic substrate was an original focus of glutamate research throughout the mid 1900s, including that of Hans Krebs, prior to later recognition of its function as an excitatory neurotransmitter [24]. We have previously suggested that in bipolar disorder “the TCA cycle undergoes several anaplerotic countermeasures [metabolic pathways that replenish citric acid cycle intermediates] to account for the lack of pyruvate oxidation…the alternative substrate glutamate is then converted to alpha ketoglutarate and reductively carboxylated through isocitrate dehydrogenase to produce citrate.” [19].

Utilization of glutamate as an alternative substrate for energy production through this mechanism typically occurs under conditions of impaired oxidative phosphorylation and increased compensatory glycolysis. Markers of increased glycolysis; elevated serum, CSF and brain lactate, have been observed consistently in MRS and metabolomics studies in bipolar disorder patients indicating a disruption of mitochondrial oxidative phosphorylation, and a switch to compensatory glycolytic energy production. In the brain, denovo glutamate is generated in astrocytes and it has been hypothesized that glutamate generated through the activity of pyruvate-carboxylase in astrocytes may account for elevated glutamate levels in bipolar disorder [25, 26].

Systematic review of biomarkers in bipolar disorder using H-MRS or 1P-MRS brain imaging also identified NAA as a significantly altered biomarker in bipolar disorder [27]. Aspartate, the precursor to NAA, is produced in the transamination reaction catalyzed by aspartate aminotransferase, in which glutamate donates its amino group to oxaloacetate, resulting in the formation of aspartate and alpha-ketoglutarate. NAA synthesis has therefore been proposed to be intricately interlinked with neuronal energetics, and glutamate oxidation in particular [28]. However, this relationship is complex, affected by many other variables and requires further research.

The same systematic review reports increased lactate as the most significantly altered biomarker in bipolar patients as detected by chromatographic, nuclear magnetic resonance, and mass spectrometry techniques [27]. Elevated lactate is a common marker of mitochondrial dysfunction, and is therefore considered an important biomarker in several mitochondrial disorders, particularly those which are accompanied by neurological symptoms [29]. Lactate is generated through glycolysis and is therefore indicative of impaired oxidative phosphorylation and increased glycolytic energy production. On this basis, it has been proposed as a marker of mitochondrial dysfunction in bipolar disorder [30]. The observations of elevated lactate in brain imaging and cerebrospinal fluid indicate a form of mitochondrial dysfunction occurring in the CNS, while similar observations in the serum may reflect either a wider systemic dysfunction or lactate exported from the CNS to the blood [27]. Extensive literature on mitochondrial dysfunction in bipolar disorder, indicating disruption of energy production in the central nervous system, further support this interpretation of lactate in bipolar disorder [5, 31–33]. Given that serum lactate is a diagnostic marker in several mitochondrial disorders, the relationship between serum and CNS lactate measures could be further explored in bipolar disorder to understand whether these may provide useful insight into the metabolic health of bipolar patients. Although there are some indications that lactate levels may vary by mood state [34], a consistent pattern linking alterations in lactate with mood has not been established.

Under these conditions of impaired energy metabolism characterized by altered glucose metabolism, reduced oxidative phosphorylation, and increased lactate, when the level of ATP in the brain falls too low, the function of ATP-dependent ion pumps and the integrity of neuronal membrane potential become compromised. In other neurological conditions, it has been observed that homeostatic mechanisms in the brain will act to provide an alternative energy substrate in response to this energy crisis [35]. Under these conditions, we propose that glutaminolysis, fueled by hyperglycolysis, occurs to restore neuronal energy, creating a state of hyper-excitability in the brain resulting in the subjective state of mania.

Under normal conditions, glutamate metabolism in the brain serves an adaptive purpose to rapidly upregulate brain metabolism in response to demanding mental tasks and challenging external stimuli [36]. Increased glycolysis and glutamate metabolism are tightly coupled in the brain. Heightened neuronal activation during mental tasks triggers an increased rate of glycolysis, along with increased activity of the glutamate-glutamine cycle to provide energy to fuel increased neuronal activity [36]. The glutamate-glutamine cycle is an important contributor to brain energy metabolism, accounting for a substantial proportion of brain glucose utilization [37]. Conversion of glutamine to glutamate in this cycle requires cytosolic reduction of NAD+ to NADH and is therefore dependent on glycolysis, which facilitates reduction of NAD+. Sibson et.al report a near-linear 1:1 relationship between glutamate neurotransmitter cycling and brain glucose metabolism, demonstrating the tight coupling of glycolysis to the glutamate-glutamine cycle [37].

There are also emerging indications that changes in glutamate metabolism may contribute to the seasonality of bipolar symptoms. A study of metabolite levels during a 30-day period around the spring equinox when risk of mania is heightened found 27 metabolites which were significantly altered in people with bipolar disorder compared with control subjects [38]. The associated pathways identified were most strongly related to glutaminolysis (conversion of glutamine to glutamate and alpha-ketoglutarate), glutamate metabolism (conversion to ammonia and L-glutamate) and arginine metabolism (involving conversion of alpha-ketoglutarate to L-glutamate).

If hyperglycolysis and glutaminolysis in the brain are a driver of mania then states akin to mania may be expected to be observed in other conditions where these states occur. Under conditions of chronic impairment of brain metabolism, such as those occurring in traumatic brain injury and other conditions discussed in this paper, we propose that similar adaptive mechanisms may be recruited to restore ATP synthesis over longer time periods with consequences such as excitotoxicity and neuronal damage.

## Hyperglycolysis and glutamate metabolism in conditions associated with bipolar disorder and secondary mania

Glutamate metabolism has been a central focus of epilepsy research, a condition which shares medications with bipolar disorder. It is also of significant interest in traumatic brain injury (TBI) research, a condition which can lead to development of bipolar disorder. Altered dynamics of glutamate metabolism and glycolysis in these conditions display cyclic or episodic occurrence and may provide insight into mechanisms relevant to BD.

The example of TBI is particularly interesting as cycling of hypometabolism and hyperglycolysis in the post-injury state is well documented and occurs as the brain progresses through several distinct neuro-metabolic states in an attempt to rescue impaired neuronal membrane potential.

The initial stage after injury is characterized by elevated extracellular potassium and intracellular calcium flux, occurring as a result of damage to lipid membranes. The subsequent sequestering of calcium into mitochondria disrupts mitochondrial function and the normal generation of ATP from glucose-derived pyruvate, creating a state of energy crisis in neurons. ATP-dependent ion pumps in neurons become unable to maintain neuronal membrane polarization and the brain reacts by inducing a state of hyperglycolysis accompanied by an increase in glutamate in an attempt to rescue neuronal membrane potential [39]. Following this state, the brain enters a hypo-metabolic state where glucose metabolism is impaired which typically lasts 7–10 days in animals and can last for several weeks in humans. The brain cycles over several weeks between these states of hypo-metabolism and hyperglycolysis as it attempts to restore ionic balance [40].

Periods of hyperglycolysis are indicated by an increase in the rate of consumption of glucose with no concomitant increase in oxidative phosphorylation [40]. This unique hypermetabolic state exceeds the brain’s capacity for oxidative phosphorylation. While this adaptation can rescue neuronal function in the short term (as a response to crisis) it is unsustainable for the brain in the long term. Similar markers to those observed in bipolar disorder are noted during this hypo/hyper-metabolic cycle: an increase in lactate and the activity of lactate dehydrogenase, reduction in NAA, and elevated glutamate [39]. It is interesting to note that psychiatric symptoms including depression and mania are observed in TBI patients during this post-injury period, and psychiatric assessments are used to measure these [41].

Glutamate metabolism in the brain has also been a focus of epilepsy research for several decades and seizures can be invoked through multiple glutamatergic mechanisms [42]. Bipolar disorder and epilepsy are significantly co-morbid and are treated by many of the same anticonvulsant medications. Similarities in the underlying pathophysiology have been described, however, there is no clear consensus on whether there are mechanisms of action of anticonvulsants which may contribute to both mood stabilization and seizure reduction [43]. Similarities in glutamate metabolism may provide some insight into this issue. For example, increased level of glutamate during seizure is accompanied by increased glycolysis, the rate of which has been observed to increase five-fold during seizure compared to normal function [44].

It is interesting to note that during these states of altered brain metabolism in the peri-ictal period, psychiatric symptoms are frequently observed. Peri-ictal mania and psychosis have been consistently documented [45, 46]. 50–60% of epilepsy patients experience psychiatric symptoms, 12% of epilepsy patients experience symptoms of bipolar disorder and about half of these have been reported to go on to be diagnosed with bipolar disorder [47, 48].

There are some areas of phenomenological overlap between bipolar symptoms and post-ictal psychiatric symptoms in epilepsy, however these are not comprehensive or consistently observed. In a detailed study of post-ictal psychiatric symptoms in epilepsy patients 88% reported post-ictal psychiatric or cognitive difficulties after more than 50% of their seizures over a 3 month period [45]. 43% reported symptoms of post-ictal depression and 22% postictal symptoms of hypomania. In this study, hypomania was defined by racing thoughts and increased energy, which have some similarity to DSM criteria for mania (racing thought and increase in goal-directed activity or psychomotor agitation). Depression was assessed by criteria including anhedonia, hopelessness, helplessness, crying bouts, feelings of self-deprecation and feelings of guilt, and these also have a degree of overlap with DSM criteria for bipolar depression (loss of interest in pleasure, depressed mood, diminished ability to think, and feelings of worthlessness or guilt). Notably the neurovegetative symptoms, experienced by 62% in this study measured by excessive somnolence, loss of appetite and loss of sexual interest also have some similarity to bipolar depression in DSM criteria (significant change in appetite and loss of interest in pleasure). While these criteria are not entirely similar, occurrence of both depression-like and hypomania-like symptoms in the post-ictal state in people with epilepsy is common and has led to the use of psychiatric medications by neurologists to manage these symptoms [49].

It is interesting to note, in the context of the shared anticonvulsant medications between bipolar disorder and epilepsy, that emerging evidence indicates that a further epilepsy treatment with effects on glutamate metabolism, ketogenic diet (KD), may show promise in treating bipolar disorder [50, 51]. We recently completed a pilot trial of a ketogenic diet for bipolar disorder and observed substantial reduction in brain glutamate measured by MRS (−13.1% in the posterior cingulate cortex (*p* < 0.001) and −9.2% in the anterior cingulate cortex (*p* = 0.02)). This was accompanied by significant correlation between daily ketone level and improved daily ecological momentary assessments of mood, energy, anxiety and impulsivity [52].

Ketone bodies, including beta-hydroxybutyrate and acetoacetate, act as alternative energy substrates in the brain and have been demonstrated to have neuroprotective effects in epilepsy and other neurological conditions. Over 100 years of clinical use of ketogenic diet and 13 randomized-controlled trials have demonstrated ketosis to be an effective therapy for seizure reduction in epilepsy [53]. A Cochrane review reports rates of seizure freedom as high as 55% and seizure reduction as high as 85% [54].

**Fig. 1:**
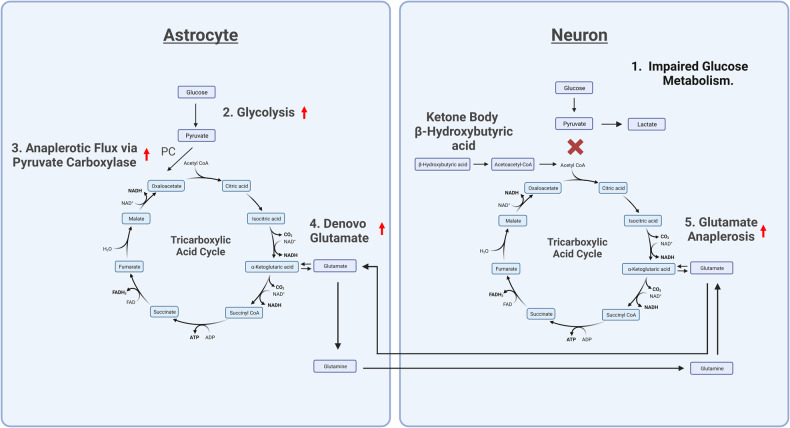
Hyperglycolysis and glutaminolysis: a response to impaired oxidative glucose metabolism. *Created with BioRender.com* 1. Impaired glucose metabolism in neurons necessitates the recruitment of an alternative fuel source for the TCA cycle. 2. In astrocytes, glycolysis metabolizes glucose to form pyruvate. 3. Pyruvate is carboxylated by the enzyme pyruvate carboxylase to form oxaloacetate in a process known as anaplerosis. 4. Oxaloacetate is then used in the TCA cycle to eventually form alpha-ketoglutarate. Alpha-ketoglutarate is converted into glutamate by a transaminase reaction. Within astrocytes, glutamate is then converted into glutamine. 5. Glutamine is released from astrocytes and taken up by neurons, where it can be converted back into glutamate. Glutamate is then converted into alpha-ketoglutarate to replenish TCA cycle intermediates and generate ATP in a process called glutaminolysis. Glucose, β-Hydroxybutyric acid, and glutamate may all serve as energy substrates for the TCA cycle via conversion to TCA cycle intermediates. Under conditions of impaired glucose metabolism, β-Hydroxybutyric acid can be used as an alternative fuel source for the TCA cycle, preventing the need for increased glutamate metabolism and avoiding associated excitotoxicity in the brain. PC pyruvate carboxylase, NAD nicotinamide adenine dinucleotide, FAD flavin adenine dinucleotide, CO_2_ carbon dioxide, ATP adenosine triphosphate, ADP Adenosine diphosphate, H_2_O dihydrogen monoxide.

Significant changes in neuronal metabolism, glycolysis, and glutamate metabolism occur in the state of ketosis. Ketone bodies can be utilized as an alternative neuronal fuel source to glucose, which bypass glycolysis as shown in Fig. 1. In this respect, they act in a similar manner to glutamate by providing an alternative energy substrate for oxidative phosphorylation. However, ketone bodies are not dependent on upregulation of glycolysis and in contrast to the excitatory properties of glutamate they may promote higher levels of inhibitory neurotransmitter GABA [55]. These distinct features of ketone bodies allow them to restore oxidative phosphorylation when glucose metabolism is impaired, without the need to induce hyperglycolysis or high levels of glutamate and excitotoxic activity in the brain [56]. In addition to ketosis induced through ketogenic diet, exogenous ketones have been demonstrated in a recent MRS study to reduce glutamate levels in the brain [57]. And in cultured neurons, ketone body beta-hydroxybutyrate reduces glutamate levels and acts as a glutamate inhibitor in NMDA receptors [58]. Ketones may, therefore, act to prevent manic episodes by removing the need for glutamate to be utilized as an alternative energy substrate when normal oxidative glucose metabolism is impaired.

Finally, it is worth noting that the brain can enter a state of increased energy demand, exceeding oxidative capacity and accompanied by upregulation of glutamate metabolism under non-pathological conditions as an adaptive response to challenging environment. Post intense exercise, an 18% increase in glutamate and glutamine and a 19% increase in lactate have been observed to meet the increased energy demands of the brain [59]. This is accompanied by heightened activation in the brain indicated by an increase in electroencephalography power across regions and frequencies [60]. Maddock et. al observed significant changes in the “oxygen-to-carbohydrate index” indicating increased uptake of glucose and lactate in the brain during and after vigorous exercise; exceeding that which can be accounted for by oxidization to CO2. These findings demonstrate that the brain generates energy from carbohydrate through non-oxidative glycolysis to supply the additional energy demand [59]. Intense exercise can be a significant trigger for hypo-mania and mania in people with bipolar disorder [61]. A study of 482 bipolar patients reported an association between frequency of exercise and number of manic episodes [62]. And this association has been reflected in qualitative interviews with patients [10]. Extensive lived experience reports of people with bipolar disorder in online fora have noted similarities in the experience of hypo-mania with the “runner’s high” experienced during and after intense exercise [63, 64].

During intense exercise, as in TBI and epilepsy, the brain experiences an acute energy demand and appears to respond by entering a state of hyperglycolysis and increased glutamate metabolism to ensure adequate fuel to the brain. During these unique metabolic states, post-concussion in TBI, in the peri-ictal period in epilepsy, and during/post intense exercise in bipolar patients, elevated mood, mania, euphoria, and manic-like symptoms have been documented. Each of these states represents periods of particular vulnerability for mania in bipolar patients.

## Future research

This hypothesis could be investigated by an examination of markers of hyperglycolysis and glutaminolysis during states of mania and euthymia in the same patients with bipolar disorder, utilizing magnetic resonance spectroscopy (MRS). ¹³C-MRS has previously been employed to measure metabolites in the glycolytic pathway and tri-citric acid cycle, and the flux of the glutamate-glutamine (glu-gln) cycle. It could therefore be utilized to investigate the relationship between these markers, cerebral neuroenergetics, and glucose metabolism [65]. Time-resolved analysis of ¹³C-MRS data has enabled tracing of the dynamic incorporation of ¹³C label into metabolites, allowing the estimation of metabolic fluxes through glycolysis and associated pathways [66]. Metabolic modeling techniques have been employed to infer the rates of glycolysis, TCA cycle, and glutamate-glutamine cycling based on ¹³C label incorporation patterns [67]. A ¹³C-MRS study combined with this analysis would allow an exploration of the relationship between glycolysis and glutaminolysis in states of mania and euthymia.

Metabolic substrates like ketone body beta-hydroxybutyrate may also be introduced through exogenous administration of ketone esters or ketone salts, and changes in these metabolites and pathways may be observed. Through this method, it may be possible to determine the relative contributions of glutamate, ketone bodies, and other metabolic substrates, such as pyruvate to the citric acid cycle. If it can be demonstrated that an increased contribution of glutamate to the citric acid cycle in bipolar disorder is displaced by beta-hydroxybutyrate, this would provide evidence in support of our hypothesis. Conversely no change in glutamate metabolism would provide evidence against our hypothesis.

Our hypothesis predicts increased activity of the glu-gln cycle in the manic state compared to euthymia. Glu-gln activity would needs to be determined alongside assessment of the activity of glutamate dehydrogenase (GDH) and aspartate aminotransferase, which facilitates the conversion of glutamate to alpha-ketoglutarate, to investigate any role of glutaminolysis in mania. Markers related to GDH, such as glutamate, alpha-ketoglutarate, ammonia (generated when GDH is deaminating glutamate and consumed when GDH is aminating α-ketoglutarate), NADH, and NADPH (produced during the deamination of glutamate and consumed during the amination of α-ketoglutarate), can be measured to reflect the redox state. Additionally, several indirect markers of glutamatergic activity including aspartate, NAA, and GABA could be investigated to provide additional metabolic context.

There are unique challenges associated with performing an MRS study on individuals experiencing a manic episode; however, the feasibility of such a study has been demonstrated previously in several cases [34, 68]. Unique considerations in methodology would be essential to ensure the safety and feasibility of such a study.

## Conclusions

The brain makes up around 2% of human body weight, and yet it sustains a remarkably high metabolic rate compared with the rest of the body, accounting for around 20% of the oxygen and therefore, calories burned [69]. Neurons remain in a state of dynamic stability and homeostasis when they have sufficient energy to sustain normal function. If energy is significantly impaired, the brain loses its equilibrium and may oscillate between states of high and low energy in an attempt to establish equilibrium. This can be observed in TBI where brain energy oscillates between periods of hypo-metabolism and hyperglycolysis in the post-concussion state.

In bipolar disorder, a less acute but chronic form of disruption of energy production may occur due to impaired insulin signaling, dysregulation of glucose metabolism and resultant mitochondrial dysfunction [70]. These metabolic perturbations act as a form of mild but persistent brain trauma occurring over long periods of time. In TBI, glycolysis is induced due to an energy deficit caused by a lack of blood supply in the damaged region and, therefore lack of oxygen for mitochondrial respiration. In bipolar disorder, glycolysis is induced due to a similar energy deficit caused by chronic impairment of oxidative glucose metabolism. We propose that this state leads to a similar oscillation between brain hypometabolism and hyperglycolysis over a longer timescale. We have noted that mania is a secondary outcome in both TBI and epilepsy and that it appears to occur during periods of energy crisis characterized by increased brain glutamate and glycolysis.

Understanding mania as a state of hyperglycolysis coupled to increased glutaminolysis may contextualize some of the commonalities between bipolar disorder and epilepsy, including the significant co-morbidity and shared medications. If ketones are made available as an alternative energy substrate to glucose, they may restore oxidative phosphorylation in neurons and limit the need for glutamate to be recruited as an alternative energy substrate. Ketones may, therefore mitigate the hypo-metabolic state by providing an alternative fuel source to glucose, restoring energy in the brain, and therefore mitigate the excitatory glutamatergic and glycolytic state (mania) by preventing the need for glutamate to be recruited as an alternative neuronal fuel source.
